# Genetic Polymorphisms of the *TGFB1* Signal Peptide and Promoter Region: Role in Wilms Tumor Susceptibility?

**DOI:** 10.15586/jkcvhl.v8i4.182

**Published:** 2021-10-16

**Authors:** Cintya Mayumi Ishibashi, Carlos Eduardo Coral de Oliveira, Roberta Losi Guembarovski, Bruna Karina Banin Hirata, Glauco Akelinghton Freire Vitiello, Alda Losi Guembarovski, Marla Karine Amarante, Karen Brajão de Oliveira, Marina Okuyama Kishima, Carolina Batista Ariza, Maria Angelica Ehara Watanabe

**Affiliations:** 1 Department of Pathological Sciences, State University of Londrina, Parana, Brazil;; 2 Department of Biology, State University of Londrina, Parana, Brazil;; 3 Department of Pathology, Clinical and Toxicological Analysis, Health Science Center, State University of Londrina, Londrina, Parana, Brazil.

**Keywords:** genetic polymorphism, nephroblastoma, prognosis, susceptibility, *TGFB1*, Wilms tumor

## Abstract

The aim of the present study was to investigate the rs1800468 (G-800A), rs1800469 (C-509T), rs1800470 (C29T), and rs1800471 (G74C) *TGFB1* genetic polymorphisms and their haplotype structures in patients with Wilms Tumor (WT) and neoplasia-free controls. The genomic DNA was extracted from 35 WT patients and 160 neoplasia-free children, and the *TGFB1* polymorphisms were genotyped by polymerase chain reaction, followed by restriction fragment length polymorphism. The haplotype structures were inferred, and permutation and logistic regression tests were performed to check for differences in haplotype distribution between the control and WT individuals. Positive associations were found in the recessive model for rs1800469 T allele (OR: 8.417; 95% CI: 3.177 to 22.297; P < 0.001) and for the rs1800470 C allele (OR: 3.000; 95% CI: 1.296 to 6.944; P = 0.01). Haplotype analysis revealed a significant negative association between GCTG and WT (OR: 0.236, 95% CI: 0.105 to 0.534; P = 0.0002); by contrast, the GTTG haplotype was associated with increased risk for WT (OR: 12.0; 95% CI: 4.202 to 34.270; P < 0.001). Furthermore, rs1800469 was negatively correlated with tumor size and a trend toward a positive correlation for capsular invasion was observed in the dominant model (Tau-b: −0.43, P = 0.02 and tau-b: 0.5, P = 0.06, respectively). This is the first study with rs1800468, rs1800469, rs1800470, and rs1800471 *TGFB1* polymorphisms in WT, and our results suggest that the *TGFB1* promoter and signal peptide region polymorphisms may be associated with WT susceptibility and clinical presentation.

## Introduction

Wilms tumor (WT), or nephroblastoma, is a childhood kidney cancer originated from the pluripotent embryonic kidney precursor (1) and consists histologically of smooth stromal, epithelial, and undifferentiated mesenchymal cells (2). It is the most common pediatric kidney tumor, affecting 1:10,000 children (3).

The incidence of this disease is increased in low-income countries, which also present the lowest survival rates for this disease. Therefore, there is a need for accurate and comprehensive records for the appropriate allocation of resources in order to improve the outcome for this curable childhood malignancy (4).

The survival rate in patients has increased considerably in recent decades to more than about 90% for localized disease and to over 70% for metastatic disease (5). However, despite a good response to therapy and high success rates, there are concerns due to the risk of irreparable side effects (6), as it targets cells with a high proliferative rate and the tumor affects tissues still in development (7). Furthermore, the prognostic indicators of recurrence and mortality are the disease stage and tumor histology, and the most significant unfavorable factors are the advanced stage and the presence of anaplasia, especially in the diffuse form, which is highly resistant to chemotherapy (8). Thus, improving our knowledge of tumor biology and biomarker identification may help to promote risk stratification and to introduce new targeted therapies that could minimize toxicity and enhance outcomes for patients with WT with unfavorable prognosis (6).

The tumor microenvironment is dynamic and its interaction with tumor cells is essential for cancer development, influencing growth, invasiveness, and metastatic process (9). In this context, transforming growth factor beta 1 (TGFβ1) is a pleiotropic cytokine that plays an important role in embryogenesis (10) and in physiological and pathological contexts by interfering with the cell cycle, apoptosis, and differentiation, also playing important roles in carcinogenic processes (11).

In normal and preneoplastic cells, TGFβ1 acts as a tumor suppressor associated with antiproliferative activity and apoptosis, but in advanced cancer stages, it acts as a tumor progression mediator (12). In malignant cells and at advanced stages of carcinogenesis, TGFβ1 promotes cell growth and epithelial-mesenchymal transition (EMT), which increase the invasiveness of these cells. In addition, it acts in the extracellular matrix remodeling and in immune system cells, inducing immunosuppression by generating regulatory T cells, anergy, and effector T-cells senescence, and angiogenesis through endothelial and smooth muscle cell activation, which favor the metastatic process (11).

In addition, it has been demonstrated that TGFβ1 regulates WT 1 gene (*WT1*) expression (13), a transcription factor necessary for kidney (14), gonads, and adrenal glands development (15). Also, its expression was found in a wide range of adult tumor types, such as colorectal cancer (16), lung cancer (17), and leukemia (18, 19).

There are also challenging aspects in WT evaluation, in relation to current and future markers of biological behavior with prognostic significance (20). Many polymorphisms have been reported in the *TGFB1* gene (21) including rs1800468 (G-800A, c.-1638G>A, c.*18G>A) and rs1800469 (C-509T, c.-1347T>C, c.*309T>C) in the promoter region and rs1800470 (c.29C>T, Leu10Pro, T869C) and rs1800471 (c.74G>C, Arg25Pro) in the signal peptide region that have been the most widely studied due to their potential functional implications on the dynamics of TGFβ1 expression and secretion. These polymorphisms have been associated with diverse cancers, including gastric cancer (22), esophageal squamous cell carcinoma (23), and breast cancer (BC) (24). Overall, results are controversial in the literature, varying according to cancer types and even among molecular subtypes and disease stage within tumor groups (24, 25). However, they have not yet been studied in WT pathogenesis.

Therefore, the aim of this study was to verify the genotype frequencies of *TGFB1* rs1800468, 1800469, 1800470, and 1800471, and their haplotype structures in WT patients and neoplasia-free controls in a Brazilian population.

## Materials and Methods

### Human subjects

The present study was approved by the Institutional Human Research Ethics Committee of Londrina State University, Paraná, Brazil (CAAE 73557317000005231). The form granting free and informed consent was signed by the parents of all the children and adolescents, and we also obtained the consent of those patients with decision-making ability to participate in the project. The WT samples consisted of 35 archived paraffin-embedded tumor tissues from child patients from the North of Paraná Laboratory of Anatomopathology and Cytology (Micropar) and the Laboratory of Pathology of the University Hospital of Londrina State University. Of these, 14 (40%) were males and 21 (60%) were females. Tumor staging distribution was 7 (29%), 6 (25%), 4 (16.7%), 6 (25%), and 1 (4.2%) for stages I, II, III, IV, and V, respectively (information was missing for 11 patients). Patients’ age ranged from 1 to 13 years (median: 3).

The control group consisted of 160 samples from neoplasia-free healthy children or young adults, which included 124 blood samples and 36 buccal cell samples collected in University Hospital of Londrina State University. Among the control group, 77 (48.1%) patients were male and 83 (51.9%) were female, and the control group age ranged from 3 months to 19 years (median: 12).

### DNA extraction

DNA from the control group was obtained from peripheral white blood cells using a Mini Spin extraction kit (Biometrix, Curitiba, Brazil), following the manufacturer’s instructions, and, in some cases, the DNA was obtained from buccal cells using a protocol based on the use of ammonium acetate (26). In the WT group, genomic DNA was isolated from formalin-fixed, paraffin-embedded tissues using the innuPREP DNA Mini Kit (Analytik Jena AG, Jena, Germany), following the manufacturer’s instructions. All DNA samples were quantified by NanoDrop 2000® Spectrophotometer (NanoDrop Technologies, Wilmington, USA) at 260nm wavelength, and the absorbance ratio at 260/280nm was used to assess protein contamination in DNA samples.

### Genetic polymorphisms genotyping

Genetic polymorphisms were analyzed by polymerase chain reaction (PCR), followed by Restriction Fragment Length Polymorphism (RFLP) analysis, as described by Jin et al. (27), with modifications. All PCR amplicons and restriction fragments were analyzed by electrophoresis in acrylamide gels (10%), detected by silver staining.

The *TGFB1* regions encompassing polymorphisms (promoter and signal peptide regions) were amplified using the same reaction condition, 4 ng/µL of DNA, 1 × high fidelity PCR buffer, 1 mM MgSO_4_, 0.2 μM primers, 0.13 mM dNTP, and 1 U Platinum^TM^ Taq DNA polymerase High Fidelity. All PCR reagents were purchased from Invitrogen ^TM^ (Carlsbad, CA, USA).

For the promoter region polymorphisms (rs1800468 and rs1800469), the primer sequences were 5’-GCAGTTGGCGAGAACAGTTG-3’ and 5’-CCAGAACGGAAGGAGAGTCAG-3’. The PCR conditions were 10 min at 94°C, 35 cycles of 45 s at 94°C, 1 min at 59°C and 1 min and 15 s at 72°C, and 10 min at 72°C. For the rs1800468 genotype analysis, PCR products (597 bp) were subjected to enzymatic digestion by *HpyCH4IV* (New England Biolabs®, Ipswich, USA) and the genotype profiles were GG (402 bp and 195 bp), GA (597 bp, 402 bp, and 195 bp), and AA (597 bp). For the rs1800469 genotype analysis, PCR products (597 bp) were subjected to digestion by *Bsu36I* (New England Biolabs^®^) and the genotype profiles were CC (488 bp and 109 bp), CT (597 bp, 488 bp, and 109 bp), and TT (597 bp).

**Figure 1:**
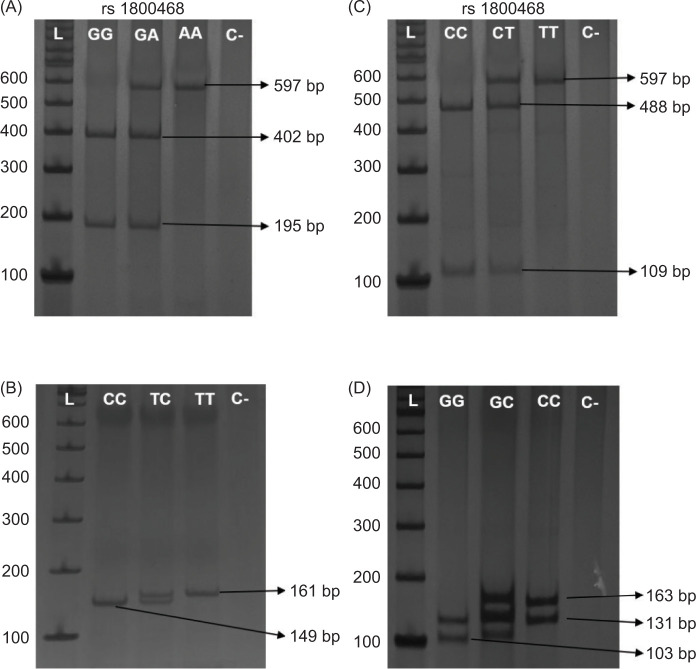
Promoter and signal peptide regions of *TGFB1* genetic polymorphisms profile. Electrophoretic profile of: (A) rs1800468 (G-800A), (B) rs1800469 (C-509T), (C) rs1800470 (c.29C>T), and (D) rs1800471 (c.74G>C). The PCR products after restriction digestion were analyzed by electrophoresis on acrylamide gel (10%), detected by silver staining method. L: Ladder 100 bp; C: negative control.

Primer sequences for the signal peptide region (rs1800470 and rs1800471) were: 5’-TTCCCTCGAGGCCCTCCTA-3’ and 5’-GCCGCAGCTTGGACAGGATC-3’ and PCR conditions were 10 min at 96°C, 35 cycles of 75 s at 96°C, 75 s at 62°C and 75 s at 73°C, and 5 min at 73°C. For the rs1800470 polymorphism, PCR products (294 bp) were subjected to enzymatic digestion by *MspA1I* (New England Biolabs®) and the genotypes were CC (149 bp, 67 bp, 40 bp, 26 bp, and 12 bp), CT (161 bp, 149 bp, 67 bp, 40 bp, and 26 bp), and TT (161 bp, 67 bp, 40 bp, and 26 bp). For the rs1800471 polymorphism, PCR products (294 bp) were subjected to enzymatic digestion by *BglI* (New England Biolabs®) and the genotype profiles were GG (131 bp, 103 bp, and 60 bp), GC (163 bp, 131 bp, 103 bp, and 60 bp), and CC (163 bp and 131 bp) (Figure 1).

To confirm the primers’ specificity used in the present study, some PCR products for both *TGFB1* regions were purified using the PureLink™ PCR Purification Kit (Invitrogen, Cashland, USA) following the manufacturer’s instructions and sequenced. The sequencing reaction was performed using the BigDye® Terminator v3.1 Cycle Sequencing Kit (Applied Biosystems®, Foster City, USA). The amplicons were sequenced in a 24-capillary 3500×l Genetic Analyzer (Applied Biosystems®). The resulting sequences revealed identity with GenBank NG_013364.1 (*TGFB1*) confirming primer specificity for all polymorphisms.

### Statistical analyses

The case-control study for WT susceptibility was performed by the Odds Ratio (OR) calculus, adopting an estimate of the relative risk at 95% confidence intervals (CI), and Fisher’s exact test. Genotypic (variant homozygotes or heterozygotes versus wild homozygotes), dominant (variant homozygotes and heterozygotes versus wild homozygotes), and recessive (variant homozygotes versus heterozygotes and wild homozygotes) models were tested for all individual polymorphisms. Correlation analyses between the polymorphisms and WT clinicopathological features were assessed by the Kendall’s tau-b rank correlation coefficient. The *TGFB1* haplotypes were determined using PHASE software version 2.1.1 (28) using all the study participants’ genotypes, and the software was used to perform permutation tests to check the difference among haplotype distributions between the control and WT individuals. All other statistical analyses were performed in IBM^®^ SPSS^®^ Statistics 24.0 *software* (IBM®, Armonk, New York, USA). All tests were two-tailed with a significance level set at 0.05.

## Results

Case-control association studies were conducted to determine the possible influence of polymorphisms on WT susceptibility. The genotype frequencies and case-control analyses for the promoter region (rs1800468 and rs1800469) and signal peptide (rs1800470 and rs1800471) polymorphisms are shown in Tables 1 and 2, respectively. The genotype frequencies for these polymorphisms in the control group were consistent with an independent control sample collected from the same geographical region (24).

**Table 1:** Genotypic frequencies in WT and control patients and case-control study for *TGFB1* promoter region polymorphisms (rs1800468 and rs1800469).

Models	Controls [n (%)]	Patients [n (%)]	Odds ratio	95% CI	P value
**rs1800468**					
GG	105 (92.1)	26 (92.8)	Reference	**–**	**–**
GA	7 (6.1)	1 (3.6)	0.577	0.068–4.898	0.577
AA	2 (1.8)	1 (3.6)	2.019	0.176–23.133	0.572
Dominant model					
GG	105 (92.1)	26 (92.8)	Reference	–	–
AA+GA	9 (7.9)	2 (7.2)	0.897	0.183–4.406	0.894
Recessive model					
GG+GA	112 (98.2)	27 (96.4)	Reference	–	–
AA	2 (1.8)	1 (3.6)	2.074	0.181–23.724	0.557
**rs1800469**					
CC	43 (37.7)	6 (24.0)	Reference	–	–
CT	58 (50.9)	6 (24.0)	0.741	0.224–2.457	0.625
TT	13 (11.4)	13 (52.0)	7.167	2.271–22.615	<0.001*
Dominant model					
CC	43 (37.7)	6 (24.0)	Reference	–	–
TT+CT	71 (62.3)	19 (76.0)	1.918	0.711–5.176	0.199
Recessive model					
CC+CT	101 (88.6)	12 (48.0)	Reference	–	–
TT	13 (11.4)	13 (52.0)	8.417	3.177–22.297	<0.001*

CI: confidence interval. Fisher’s exact test. *Significant (P < 0.05).

**Table 2:** Genotypic frequencies in WT and control patients and case-control study for *TGFB1* signal peptide region polymorphisms (rs1800470 and rs1800471).

Models	Controls [n (%)]	Patients [n (%)]	Odds ratio	95% CI	P value
**rs1800470**					
TT	24 (16.7)	12 (37.5)	Reference	–	–
TC	83 (57.6)	11 (34.4)	0.545	0.208–1.426	0.216
CC	37 (25.7)	9 (28.1)	2.056	0.752–5.618	0.160
Dominant model					
TT	24 (16.7)	12 (37.5)	Reference	–	–
CC+TC	120 (83.3)	20 (62.5)	0.884	0.375–2.081	0.777
Recessive model					
TT+TC	107 (74.3)	23 (71.9)	Reference	–	–
CC	37 (25.7)	9 (28.1)	3.000	1.296–6.944	0.01*
**rs1800471**					
GG	130 (90.3)	26 (81.2)	Reference	–	–
GC	14 (9.7)	3 (9.4)	1.071	0.287–3.995	0.918
CC	–	3 (9.4)	NA	NA	NA
Dominant model					
GG	130 (90.3)	26 (81.2)	Reference	–	–
CC+GC	14 (9.7)	6 (18.8)	2.143	0.754–6.093	0.153

CI: confidence interval; Fisher’s exact test; *Significant (P < 0.05); NA: not available.

For the *TGFB1* promoter region polymorphisms, the case-control study indicated no significant association for the rs1800468 polymorphism. However, the rs1800469 polymorphism presented a positive association for the T allele in the recessive model (OR: 8.417; 95% CI: 3.177 to 22.297; P < 0.001) (Table 1).

Similarly, when the *TGFB1* signal peptide polymorphisms were analyzed, a significant association was found for the rs1800470 polymorphism with increased risk for WT in the recessive model (OR: 3.000; 95% CI: 1.296 to 6.944; P = 0.01). However, no significant association was found for the rs1800471 polymorphism (Table 2). For this polymorphism, analysis evaluating the CC genotype was not possible due to the absence of this genotype in the control group.

Correlation between polymorphisms and clinicopathological features were analyzed considering additive, dominant, and recessive models. A negative correlation was observed between tumor size (≤8 cm vs >8 cm) and rs1800469 in the dominant model (Tau-b = −0.43; P = 0.02; Table 3). Furthermore, the lack of statistical significance in the positive correlation between this model and capsular invasion was only marginal (Tau-b = 0.5; P = 0.06; Table 3). No other significant correlation was observed for any variant analyzed.

**Table 3:** Correlation analyses between *TGFB1* rs1800469 and WT patient’s clinicopathological features.

Parameter	C-509T genotypes [n (%)]			Models (Tau-b; P)		
	CC	CT	TT	Additive	Dominant	Recessive
**Tumor size**						
≤8 cm	0 (0.0)	3 (60.0)	2 (40.0)	−0.12; 0.61	−0.43; 0.02*	0.09; 0.71
>8 cm	4 (40.0)	1 (10.0)	5 (50.0)			
**Capsular invasion**						
Absent	3 (60.0)	0 (0.0)	2 (40.0)	0.405; 0.13	0.50; 0.06	0.35; 0.20
Present	1 (12.5)	1 (12.5)	6 (75.0)			
**Nodal involvement**						
Absent	3 (37.5)	1 (12.5)	4 (50.0)	−0.07; 0.77	0.04; 0.87	−0.17; 0.52
Present	2 (33.3)	2 (33.3)	2 (33.3)			
**Distant metastasis**						
Absent	1 (33.3)	1 (33.3)	1 (33.3)	0.00; 1.00	0.00; 1.00	0.00; 1.00
Present	2 (33.3)	2 (33.3)	2 (33.3)			

* Significant (P < 0.05).

Twenty-two WT samples that could be genotyped for the four polymorphisms were included for haplotype analysis. Nine possible inferred haplotypes were observed (Table 4). A significant difference among the controls and patients with WT was observed (P = 0.001) in global haplotype distribution (Table 4). Based on these results, a case-control study of the association of individual haplotype structures indicated that the GCTG haplotype conferred protection against tumor development (OR: 0.236; 95% CI: 0.105 to 0.534; P = 0.0002) and GTTG was associated with risk (OR: 12.000; 95% CI: 4.202 to 34.270; P < 0.0001) (Table 4). For this analysis, each haplotype structure was compared to the total number of haplotypes in the group, and the ACCC and ATCG haplotype structures were not considered due to their absence in the control group.

**Table 4:** Case-control association study for haplotype structures.

Haplotype structure	Control [n (%)]	WT [n (%)]	Odds ratio (CI)	P value
ACCC	0 (0.0)	1 (2.3)	NA	–
ACTG	10 (5.0)	1 (2.3)	0.437 (0.055–3.509)	0.694
ATCG	0 (0.0)	1 (2.3)	NA	–
GCCC	7 (3.5)	2 (4.6)	1.049 (0.212–5.206)	1.000
GCCG	14 (7.1)	2 (4.6)	0.506 (0.111–2.297)	0.534
GCTG	96 (48.5)	8 (18.2)	0.236 (0.105–0.534)	<0.001*
GTCC	3 (1.5)	1 (2.3)	1.512 (0.153–14.890)	0.554
GTCG	62 (31.3)	16 (36.4)	1.253 (0.635–2.484)	0.593
GTTG	6 (3.0)	12 (27.3)	12.000 (4.202–34.270)	<0.001*
Permutation P-value	<0.001*		–	–

CI: confidence interval. NA: not analyzed. *Significant (P < 0.05) through permutation analysis (for global haplotype distribution) or Fisher’s exact test (for odds ratio).

## Discussion

WT is a neoplasm of embryonic origin whose structures and composition recapitulate characteristics of normal nephrogenesis (29). Failures during embryogenesis are considered as the causes of WT (1). In this context, studies have demonstrated the crucial role of the TGFβ1 in metanephric development during the fetal period (10), as well as in immunomodulation by inhibiting the activity of essential immune cells for the antitumor response. Thus, it is reasonable to assume that TGFβ1 also participates in WT development (30).

In the present study, we investigated four polymorphisms in *TGFB1*, two in the promoter region (rs1800468 and rs1800469) and two in the signal peptide region (rs1800470 and rs1800471), that are somehow involved in expression regulation and secretion of this cytokine, in relation to WT susceptibility.

Despite numerous studies describing a role for TGFβ1 in various neoplasms (22, 31), only two have demonstrated the direct participation of this cytokine in WT, associating TGFβ1 expression in tumor microenvironment invasion and disease progression (32), and showing that TGFβ1 signaling is the most important coordinator of anaplastic histology (33). However, studies on WT and genetic polymorphisms are even more scarce, and this is the first study evidencing these *TGFB1* polymorphisms in this disease.

In this study, no association was found for the rs1800468 polymorphism of the *TGFB1* promoter region. Although no studies have evaluated this polymorphism in WT, some studies on other tumors have demonstrated increased risk associated with the A allele, such as BC (34) and uterine cervical cancer (35), while others failed to observe any significant association (24, 27).

Nevertheless, when the rs1800469 *TGFB1* polymorphism was analyzed, the T allele was associated with WT susceptibility in the recessive model. While Jin, Deng (23), in a study on esophageal squamous cell carcinoma, have found association of the same variant with protection against cancer, studies on BC (24, 34) differently have shown an association of rs1800469 T allele with the risk of cancer.

The *TGFB1* rs1800469 polymorphism is located in a Yin Yang 1 (YY1) transcription factor consensus-binding site. Studies using transient transfection analysis with promoter–reporter constructs showed that the exchange from C to T at the −509 position of the promoter region could increase affinity for YY1 (36). This may be one of the mechanisms that leads to increased cytokine secretion in cancer patients’ plasma, as observed in cases of pancreatic cancer (31), gastric cancer (22), as well as in cases of WT with poor prognosis (32).

For the signal peptide region, it was verified that the rs1800470 C allele was associated with WT development, in a recessive model. Similar results were found for other tumors, in which homozygous CC was associated with BC (37) and colorectal cancer (38) susceptibility.

Significant effect of rs1800470 polymorphism on the TGFβ1 secretion may provide an explanation for the reported associations with a variety of diseases, such as that of the present study. This polymorphism results in the exchange of a leucine (T) for a proline (C) at signal peptide amino acid 10, and it has been reported previously that the amount of TGFβ1 in serum is higher for CC homozygotes than TT homozygotes (39). Dunning, Ellis et al. (37), in a transfection study of HeLa cells with constructs encoding either the C or T allele of *TGFB1*, indicated that the signal peptide with the C allele causes a 2.8-fold increase in secretion compared with the T allele form. This may reflect structure and property changes in the TGFβ1 signal peptide due to the substitution of amino acids.

Susianti, Handono (40) showed that the presence of proline amino acid (allele C) changes the hydrophobic core region of the TGFβ1 signal peptide and breaks the α-helix structure favored by leucine (allele T), altering the stability of the signal peptide interaction with the Signal Recognition Particle (SRP) and translocation complex in endoplasmic reticulum, reducing the values on transmembrane tendency, and stabilizing protein partner binding. As the sequence of the signal peptide is responsible for allowing the export of new proteins to endoplasmic reticulum lumen and then secretion, such changes could modify the exportation and secretion dynamics, with consequent cytokine-level alteration.

It is therefore plausible that TGFβ1 local secretion by tumors and/or local stromal cells is also higher for CC homozygotes. As noted earlier, current hypotheses on the effects of TGFβ1 on tumor development suggest that increased amounts of this cytokine activity may suppress the early stages of tumor development but promote the invasiveness, metastasis, and angiogenesis of advanced tumors (37). In this context, Zhang, Liu (32) reported that TGFβ1 expression in WT tissues was associated with invasion/metastasis, confirmed by an invasion assay through transiently transfected *TGFB1* to primary WT cells.

Moreover, Hamatani et al. (13) verified that long-term TGFβ1 stimulation altered the methylation pattern of regulatory regions and decreased *WT1* expression in a human podocyte cell line. The WT1 protein is a transcription factor, which plays multiple roles in development, including kidney development, tissue homeostasis, and disease (41). *WT1* was initially discovered as a tumor suppressor in WT (42) and the first gene found to be inactivated in WT (1). Our results indicated the association of two *TGFB1* polymorphisms, which lead to increased cytokine secretion, with the development of WT. As observed by Hamatani et al. (13), overexpression of TGFβ1 leads to under expression of WT1, and this could be a mechanism involved in WT development.

In this study, no association was found between rs1800471 signal peptide polymorphism and WT susceptibility, as described in bladder (43) and colorectal adenoma cancers (38).

In clinicopathological analyses, rs1800469 in the dominant model indicated smaller tumor size and a trend toward a positive correlation was shown for capsular invasion. This might indicate the potential action in TGFβ1 cytostatic effect in controlling cell proliferation but mediating EMT and invasion, as previously reported in WT (30, 32, 33). The dysregulation of TGFBβ has been linked to the initiation and progression of multiple human cancers, including WT (44).

Several diseases have been found at higher frequencies in individuals with haplotypes of certain genes, but there are no studies on WT. The *TGFB1* haplotype association analysis revealed that the GCTG structure conferred protection against WT while the GTTG haplotype, which differs only by rs1800469, confers risk. Although the individual C allele of rs 1800470 polymorphism conferred risk for WT, this was not observed in the haplotype analysis, perhaps due to the small number of samples included in the haplotype analysis (22 samples). Moreover, here we showed a positive association for the T allele (rs1800469) and C allele (rs1800470) with WT susceptibility, although a larger number of samples is necessary to confirm this potential association.

Few studies have performed a haplotype analysis of *TGFB1* polymorphisms. Jin, Hemminki (27) conducted an analysis with the same four *TGFB1* polymorphisms (promoter and signal peptide regions) but no associations with BC were found; on the other hand, our group has showed subtype-specific associations between *TGFB1* haplotypes and BC risk (24). Berndt et al. (38), in a study with five *TGFB1* polymorphisms (rs1800468, rs1800469, rs1800470, rs1800471, and rs1800472), found an association of the GTCGC haplotype structure with susceptibility to colorectal adenoma, which is partially compatible with our individual polymorphism analysis, where the T allele of rs1800469 (C-509T) and C allele of rs1800470 (T869C) polymorphism conferred risk for WT.

## Conclusion

This is the first study with the rs1800468, rs1800469, rs1800470, and rs1800471 *TGFB1* polymorphisms in WT patients, and our results suggest that the rs1800469 and rs1800470 polymorphisms may serve as markers associated with the susceptibility and clinical presentation of this disease.
